# How satellite InSAR has grown from opportunistic science to routine monitoring over the last decade

**DOI:** 10.1038/s41467-020-17587-6

**Published:** 2020-08-13

**Authors:** Juliet Biggs, Tim J. Wright

**Affiliations:** 1 COMET, School of Earth Sciences, University of Bristol, Bristol, BS8 1TH UK; 2 COMET, School of Earth and Environment, University of Leeds, Leeds, LS2 9JT UK

**Keywords:** Natural hazards, Seismology, Volcanology

## Abstract

In the past decade, a new generation of radar satellites have revolutionised our ability to measure Earth’s surface deformation globally and with unprecedented resolution. InSAR is transforming our understanding of faults, volcanoes and ground stability and increasingly influencing hazard management.

## A brief history of satellite-based radar interferometry

**Fig. 1:**
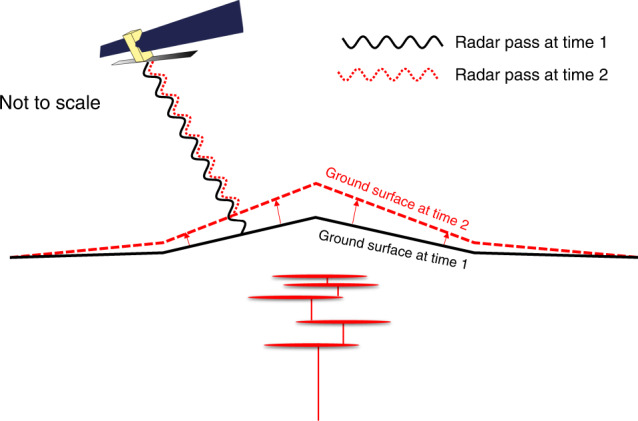
Measuring surface movement with InSAR. An orbiting satellite sends a coherent radar signal to the surface and measures the backscattered radiation. The phase difference (position in the wave cycle) between the signals returning at two different times (time 1 in black and time 2 in red) can be used to estimate ground movement caused by a range of mechanisms.

The theory of Interferometric Synthetic Aperture Radar (InSAR) has been known for decades: after applying various geometric and atmospheric corrections, high resolution maps of surface displacements can be produced by comparing the phase of successive radar images (Fig. 1). The first practical demonstration was in 1992, when the ERS-1 satellite captured surface deformation caused by the Landers, California earthquake^1^. The image featured on the cover of Nature and the results inspired a generation of scientists. Since then, the quality and quantity of available images has increased dramatically. The last decade has seen the first mission specifically designed for ground deformation monitoring (European Union’s Sentinel-1 constellation), a global Digital Elevation Model with unprecedented accuracy (TanDEM-X), and a constellation of small SAR satellites capable of acquiring imagery with 1 m resolution (CosmoSkyMed). These satellites, and others, now measure ground motion across the planet several times per day and can achieve accuracy of less than a millimetre per year allowing us to better understand the processes by which the Earth deforms and to provide relevant information for disaster risk reduction.

## Understanding tectonics and mitigating seismic hazards

**Fig. 2:**
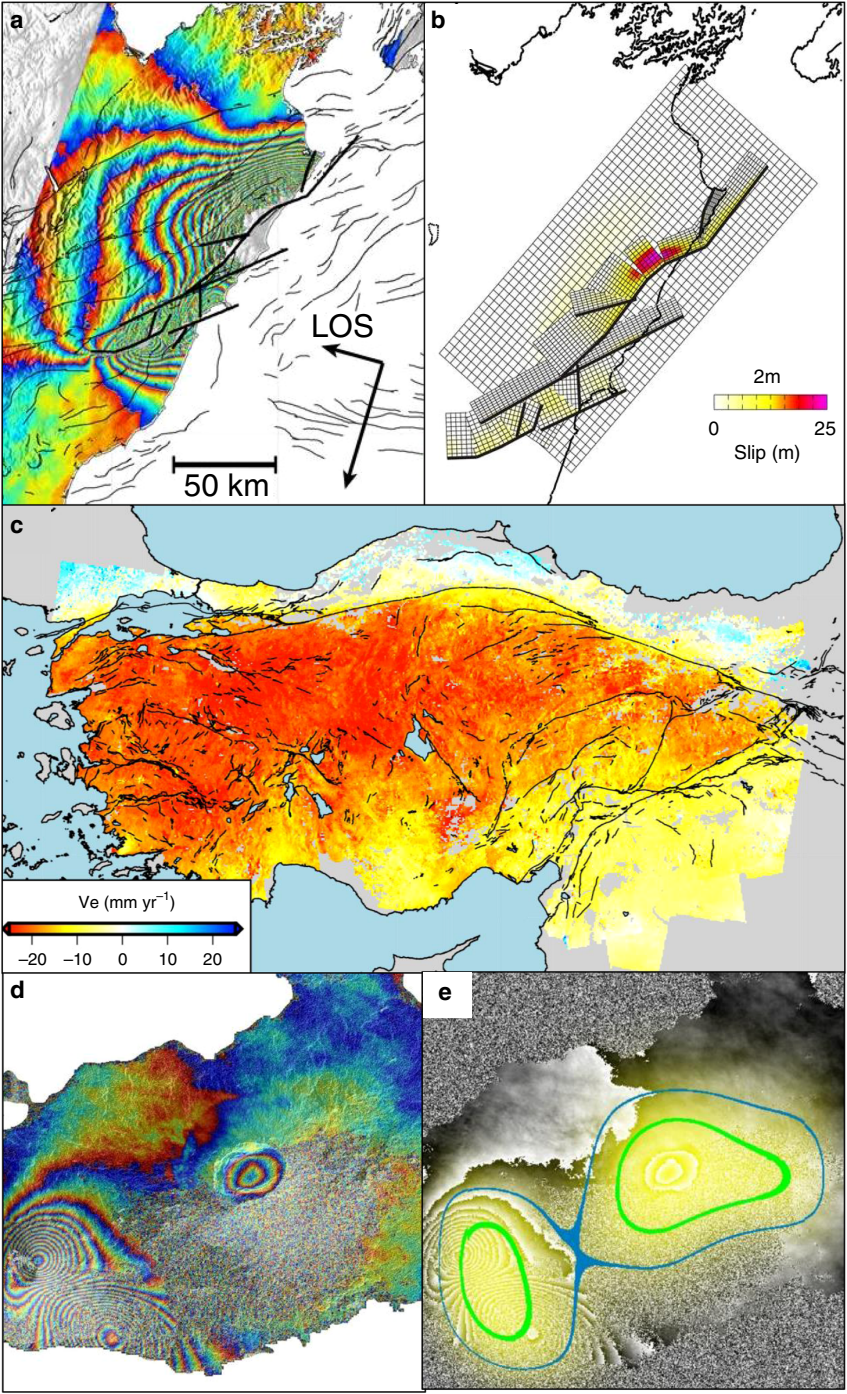
Examples of InSAR applied to earthquakes, tectonics and volcanism. **a** ALOS-2 image showing the line-of-sight (LOS) ground motion during the 2016 Kaikōura earthquake, New Zealand. Each coloured fringe corresponds to 11.4 cm of deformation. Heavy black lines show the faults that slipped. From^4^ **b** Best-fitting slip model for the 2016 Kaikōura earthquake, including slip on the subduction interface and at least 12 major structural faults. From^4^ **c** East-west velocity of the Anatolian microplate derived from the first 5 years of Sentinel data combined with ground-based GNSS measurements from ref. ^9^. Black lines are active faults from ref. ^20^. **d** Sentinel-1 image showing a magmatic intrusion on the flanks of Sierra Negra volcano, Galapagos and subsidence at the summit. Each coloured fringe corresponds to 2.8 cm of motion in the satellite line-of-sight. **e** The image in **d** classified using the convolutional neural network of ref. ^19^ to automatically identify areas of deformation. Contours show probabilities of 0.5 and 0.8, respectively.

Since the early 20^th^ Century we have understood that earthquakes occur because of the sudden, catastrophic release of strain energy that has slowly accumulated around tectonic faults^2^. However, at the time of the 1992 Landers earthquake, we had only measured the resultant coseismic deformation for a handful of earthquakes. InSAR data, mostly acquired by the European Space Agency’s (ESA) European Remote Sensing (ERS) and Envisat satellites in the 1990s and 2000s, have now allowed scientists to image deformation from more than 130 earthquakes^3^, a number that is increasing by 20–30 events per year thanks to systematic global acquisitions from Sentinel-1. These data have been used to help constrain detailed models of the fault slip that caused the earthquakes, confirming that the Earth does behave, as expected, like an elastic solid. However, InSAR also shows precisely which faults slipped during an event; and we have been surprised by how often earthquakes occur in unexpected locations and the complexity of fault ruptures. The best example of this surprising complexity is the 2016 Kaikoura Earthquake in New Zealand, which ruptured a network of a dozen or more faults^4^ (Fig. 2a,b). InSAR data, combined with seismology, has also shown that fault ruptures can also jump, with triggered fault slip (seismic and aseismic) occurring at large distances from the initial ruptures. This complexity is now being factored into future seismic hazard models for urban areas like Los Angeles at risk from complex, jumping, multi-fault ruptures. The InSAR data are also helping us interpret the geomorphological records of fault motion—we have learned, for example, that slip at the surface is not necessarily a good guide to what happened at depth^5^.

Between earthquakes, InSAR studies have revealed that faults undergo a wide range of aseismic phenomena (those that occur without radiating significant seismic waves). InSAR has been used to identify aseismic slow earthquakes (transient creep events) on both continental faults^6^ and subduction megathrusts^7^. Following earthquakes we typically see aseismic deformation transients, which can last several decades; these occur in response to coseismic stress changes and have been attributed to a combination of continued slip on the fault plane, fluid flow in the porous rocks surrounding the fault, and viscous relaxation of the hot rocks in the lower crust and mantle^8^. By capturing the details of these aseismic transients we can use the geodetic data to place constraints on the frictional properties of faults and the rheology of the crust and mantle.

A major driver for these studies is to say something about where and when future earthquakes might occur. While short-term prediction of earthquakes is extremely challenging, if not impossible, we do expect most earthquakes to be preceded by the long-term accumulation of strain energy. If we can map the spatial distribution and rate of this strain accumulation, then this should help refine long-term forecasts of earthquake rates. InSAR data are now routinely used alongside Global Navigation Satellite Systems data (like GPS) to map interseismic strain over large regions^9^ (Fig. 2c). Although the data typically record only 5–10 years of deformation so far, this may be representative of the long-term rate of strain accumulation, at least for large faults like the North Anatolian Fault^10^. In the near future, as long time series of data from Sentinel-1 and other systems reduce measurement uncertainties, we should see high-resolution maps of tectonic strain accumulation in the tectonic belts derived from InSAR and GNSS, and these incorporated into future models of seismic hazard^11^.

## Understanding magmatism and mitigating volcanic hazards

Another source of surface movement is magma moving underground: visual observations of bulges or changes in relative sea level preceding eruptions have been reported for centuries, and ground-based surveying techniques measured deformation at a handful of well-studied volcanoes during the 20^th^ century. However, it was not until the satellite era that is was possible to measure remote or dangerous volcanoes, or to survey large regions^12^ (Fig. 2d). The past decade has seen InSAR images of some dramatic events such as 500 m of caldera collapse in Kilauea, Hawaii^13^. Ongoing improvements in satellite technology and data processing have reduced detection thresholds while increasing coverage, causing a dramatic rise in the number of volcanoes known to be deforming and providing the first detailed information on the spatial and temporal characteristics of volcanic deformation. The first satellite-based catalogue recorded 336 deformation events at 160 different volcanoes, revealing a remarkable diversity in the patterns, rates, durations and extents^14^. Statistical analysis shows that roughly half of these deformation events are linked to eruptions^15^ and satellite data is increasingly used by volcano observatories. Perhaps the best example of the use of satellite data for decision-making is the radar observation of rapid dome growth at Merapi, Indonesia – the subsequent decision to evacuate an additional 400,000 people is credited with saving 10,000–20,000 lives^16^.

InSAR data, together with information from geochemistry, petrology, and other geophysical methods, are contributing to a multidisciplinary paradigm shift in our conceptual understanding of magmatic systems^17^. The simple model of liquid magma pressurising an elastic-sided chamber until failure can no longer explain the wealth of observations. Instead, the prevailing view is of a transcrustal system composed of multiple lenses of crystals, melt and gases^17^. Translating this new view of magmatic systems to the interpretation of volcano deformation will require a multidisciplinary approach, but through improved eruption forecasts, will ultimately benefit the 800 million people globally who live within 100 km of a volcano.

## Monitoring anthropogenic surface motion and outlook

The growth of satellite deformation data has made us increasingly aware that human activity also causes surface motion. Fluids, including hydrocarbons and ground water, are extracted from and injected into the subsurface by various industries, while poorly-constructed buildings and infrastructure can deform and collapse. Even in the UK, where tectonic activity is low, the average cost of ground movement is estimated to be £300–500 million per year, while a single earthwork failure on a mainline railway could cost over £10M^18^. Now that Sentinel-1 is providing a reliable and long-term archive of InSAR ground measurements, satellite data will likely play an increasing role in monitoring and mitigating against the impacts of anthropogenic sources of ground motion. Several national ground motion products are already available and pan-national services are under development with potential impacts on the civil, geotechnical and structural engineering sectors.

By combining data from several existing SAR satellites, daily monitoring of ground motion is now possible, whether that be the deformation caused by an earthquake, the evolution of the magmatic system during a volcanic crisis, or the failure of an embankment. In the future, InSAR monitoring will become ever more routine, and the launch of several new SAR missions with new capabilities is already scheduled. Handling the large volumes of data generated is already challenging, and machine learning algorithms^19^ are likely to be important for maximising the benefit from the data (Fig. 2e). Over the last 10 years InSAR has moved from being a niche research curiosity to a global monitoring tool with enormous potential. The next 10 years will likely see InSAR analysed alongside other satellite and ground based observations and machine learning algorithms to provide critical tools to help us live our lives safely and without disruption on our dynamic, unstable and dangerous planet.

## References

[1] Massonnet D (1993). The displacement field of the Landers Earthquake mapped by radar interferometry. Nature.

[2] Reid, H. F. *The mechanics of the earthquake: the California earthquake of 18 April, 1906. Report of the State Earthquake Investigation Commission, no. 2*., (Carnegie institution of Washington, 1910).

[3] Funning GJ, Garcia A (2018). A systematic study of earthquake detectability using Sentinel-1 Interferometric Wide-Swath data. Geophys. J. Int..

[4] Hamling, I. J. et al. Complex multifault rupture during the 2016 Mw 7.8 Kaikōura earthquake, New Zealand. *Science* **356**, 10.1126/science.aam7194 (2017). 10.1126/science.aam7194 28336563

[5] Kaneko Y, Fialko Y (2011). Shallow slip deficit due to large strike-slip earthquakes in dynamic rupture simulations with elasto-plastic off-fault response. Geophys. J. Int..

[6] Khoshmanesh, M. & Shirzaei, M. Episodic creep events on the San Andreas Fault caused by pore pressure variations. *Nat. Geosci.*, 10.1038/s41561-018-0160-2 (2018). 10.1038/s41561-018-0160-2 PMC6008793 29937919

[7] Bekaert D, Hooper A, Wright T (2015). Reassessing the 2006 Guerrero slow‐slip event, Mexico: Implications for large earthquakes in the Guerrero Gap. J. Geophys. Res..

[8] Wright TJ, Elliott JR, Wang H, Ryder I (2013). Earthquake cycle deformation and the Moho: Implications for the rheology of continental lithosphere. Tectonophysics.

[9] Weiss, J. R. et al. High‐resolution surface velocities and strain for Anatolia from Sentinel‐1 InSAR and GNSS data. *Geophys. Res. Lett.* e2020GL087376 (2020).

[10] Hussain E (2018). Constant strain accumulation rate between major earthquakes on the North Anatolian Fault. Nat. Commun..

[11] Bird P, Kreemer C (2015). Revised tectonic forecast of global shallow seismicity based on version 2.1 of the Global Strain Rate Map. Bull. Seismol. Soc. Am..

[12] Pritchard ME, Simons M (2002). A satellite geodetic survey of large-scale deformation of volcanic centres in the central Andes. Nature.

[13] Anderson, K. R. et al. Magma reservoir failure and the onset of caldera collapse at Kīlauea Volcano in 2018. *Science* **366**, eaaz1822 (2019). 10.1126/science.aaz1822 31806783

[14] Ebmeier S (2018). Synthesis of global satellite observations of magmatic and volcanic deformation: implications for volcano monitoring & the lateral extent of magmatic domains. J. Appl. Volcanol..

[15] Biggs, J. et al. Global link between deformation and volcanic eruption quantified by satellite imagery. *Nat. Commun.* **5**, 10.1038/ncomms4471 (2014). 10.1038/ncomms4471 PMC4409635 24699342

[16] Pallister J (2013). Merapi 2010 eruption—Chronology and extrusion rates monitored with satellite radar and used in eruption forecasting. J. Volcanol. Geotherm. Res..

[17] Cashman KV, Sparks RSJ, Blundy JD (2017). Vertically extensive and unstable magmatic systems: a unified view of igneous processes. Science.

[18] Pritchard, O. G., Hallett, S. H. & Farewell, T. S. Soil movement in the UK–Impacts on critical infrastructure. *Infrastructure Transitions Research Consortium* (Cranfield University, Cranfield, 2013).

[19] Anantrasirichai N, Biggs J, Albino F, Bull D (2019). A deep learning approach to detecting volcano deformation from satellite imagery using synthetic datasets. Remote Sens. Environ..

[20] Emre Ö (2018). Active fault database of Turkey. Bull. Earthq. Eng..

